# MiR-221-regulated KIT level by wild type or leukemia mutant RUNX1: a determinant of single myeloblast fate decisions that – collectively – drives or hinders granulopoiesis

**DOI:** 10.18632/oncotarget.21266

**Published:** 2017-09-23

**Authors:** Stefano Rossetti, Michael J. Anauo, Nicoletta Sacchi

**Affiliations:** ^1^ Department of Cancer Genetics and Genomics, Roswell Park Cancer Institute, Buffalo, NY 14263

**Keywords:** wild type RUNX1, RUNX1-MTG8 mutant, miR-221-KIT axis, miR-ON reporter, single myeloblast fate decisions

## Abstract

RUNX1, a master transcription factor of hematopoiesis, was shown to orchestrate both cell proliferation and differentiation during granulopoiesis by regulating microRNAs (miRs). In this study, taking advantage of the miR-ON reporter system, we monitored first, how the granulocyte colony stimulation factor (GCSF) temporally modulates the concomitant level variation of miR-221 and one of its prototypic targets, the stem cell factor receptor KIT, in single 32D^miR-ON-221^ myeloblasts expressing wild type RUNX1. Second, with the same reporter system we assessed how these temporal dynamics are affected by the t(8;21)(q22;q22) acute myelogenous leukemia mutant RUNX1-MTG8 (RM8) in single 32D-RM8^miR-ON-221^ myeloblasts. Depending on either wild type, or mutant, RUNX1 transcriptional regulation, the cell-context specific miR-221-regulated KIT level translates into differential single cell fate decisions. Collectively, single cell fate choices translate into either initial expansion of undifferentiated myeloblasts followed by terminal granulocyte differentiation, as it happens in normal granulopoiesis, or aggressive growth of undifferentiated myeloblasts, as it happens in RUNX1-MTG8-positive acute myelogenous leukemia. Increasing knowledge of biological changes, due to altered miRNA dynamics, is expected to have relevant translational implications for leukemia detection and treatment.

## INTRODUCTION

Cell proliferation and differentiation are intimately linked processes of normal embryonic and postembryonic development and tissue homeostasis [1–3]. In hematopoiesis, a major determinant of cell fate decisions is RUNX1, the alpha subunit of the Core Binding Factor (CBF) [4–6]. RUNX1, as heterodimer with the CBF beta (CBFB) subunit, epigenetically regulates the transcription of a myriad of coding and non-coding RUNX1-target genes [7–9].

A prototypic non-coding RUNX1-target gene is miR-221, which plays a key role during granulopoiesis by regulating the expression level of the KIT receptor [10, 11]. KIT is a cell surface tyrosine kinase receptor that, upon binding the stem cell factor (SCF), activates signaling pathways of cell survival and proliferation [12].

Previously, using the 32D mouse myeloid progenitor model, we found that incremental *exogenous* KIT expression delays granulocytic differentiation in response to granulocyte colony stimulating factor (GCSF), by promoting cell proliferation both in a time- and GCSF-dose-dependent manner [13]. Moreover, inhibition of *exogenous* KIT-mediated proliferation with an inhibitor of KIT activity (Imatinib) was shown to enable GCSF-induced 32D granulocytic differentiation [13].

In this study we instead show that GCSF-induced upregulation of *endogenous* KIT occurs in a) undifferentiated 32D myeloblasts expressing wild type RUNX1, with the potential of maturing into differentiated granulocytes as well as in b) undifferentiated 32D myeloblasts expressing the RUNX1-MTG8 (also AML1-ETO or RUNX1T1-RUNX1, and here abbreviated as RM8) which are capable only of continuous growth, as it happens in the t(8;21)(q22;q22) acute myelogenous leukemia (AML). In order to test the contribution of single cells to either the temporal shift from myeloblast proliferation to granulocytic differentiation in a 32D population of cells expressing wild type RUNX1, or the increasing proliferation potential of a 32D population of cells expressing the RM8 mutant, we set out to concomitantly assess the temporal level variation of *endogenous* miR-221-regulated KIT in single cells.

Methods for detecting miRNAs in live cells include the color-tunable molecular beacon method [14], the RNAi-Inducible Luciferase Expression System (RILES) [15], and the miR-ON reporter system, which allows miRNA expression quantification based on green fluorescent protein (GFP) expression in single cells [16]. By using the miR-ON reporter system, we could assess the concomitant temporal variation of both miR-221 and KIT levels either in single 32D^miR-ON-221^ cells with wild type RUNX1 or in single 32D-RM8^miR-ON-221^ cells with the RM8 mutant. With this strategy we found evidence that GCSF-induced cell proliferation delays 32D^miR-ON-221^ granulocytic differentiation depending on the collective contribution of RUNX1-regulated miR-221 level in single cells, which in turn, determines the endogenous KIT level in each cell. In contrast, we found that GCSF-induced proliferation of 32D-RM8^miR-ON-221^ cells, collectively due to progressive single cell context-specific miR-221 transcriptional repression, by leading to progressive single cell increase of KIT upregulation, hampers granulocytic differentiation.

As shown here, depending on either normal or mutant RUNX1, cell-wise miR-221-regulated KIT level translates into individual different cell decisions. However, collectively, individual cell decisions lead to either initial expansion of wild type RUNX1 undifferentiated myeloblasts, followed by terminal granulocyte differentiation, as it happens in normal granulopoiesis, or incremental proliferation of undifferentiated RM8 myeloblasts, as it happens in t(8;21) acute myelogenous leukemia.

## RESULTS

### Evidence of antithetic variation of miR-221 and KIT levels in single 32D^miR-ON-221^ cells during granulopoiesis

**Figure 1:**
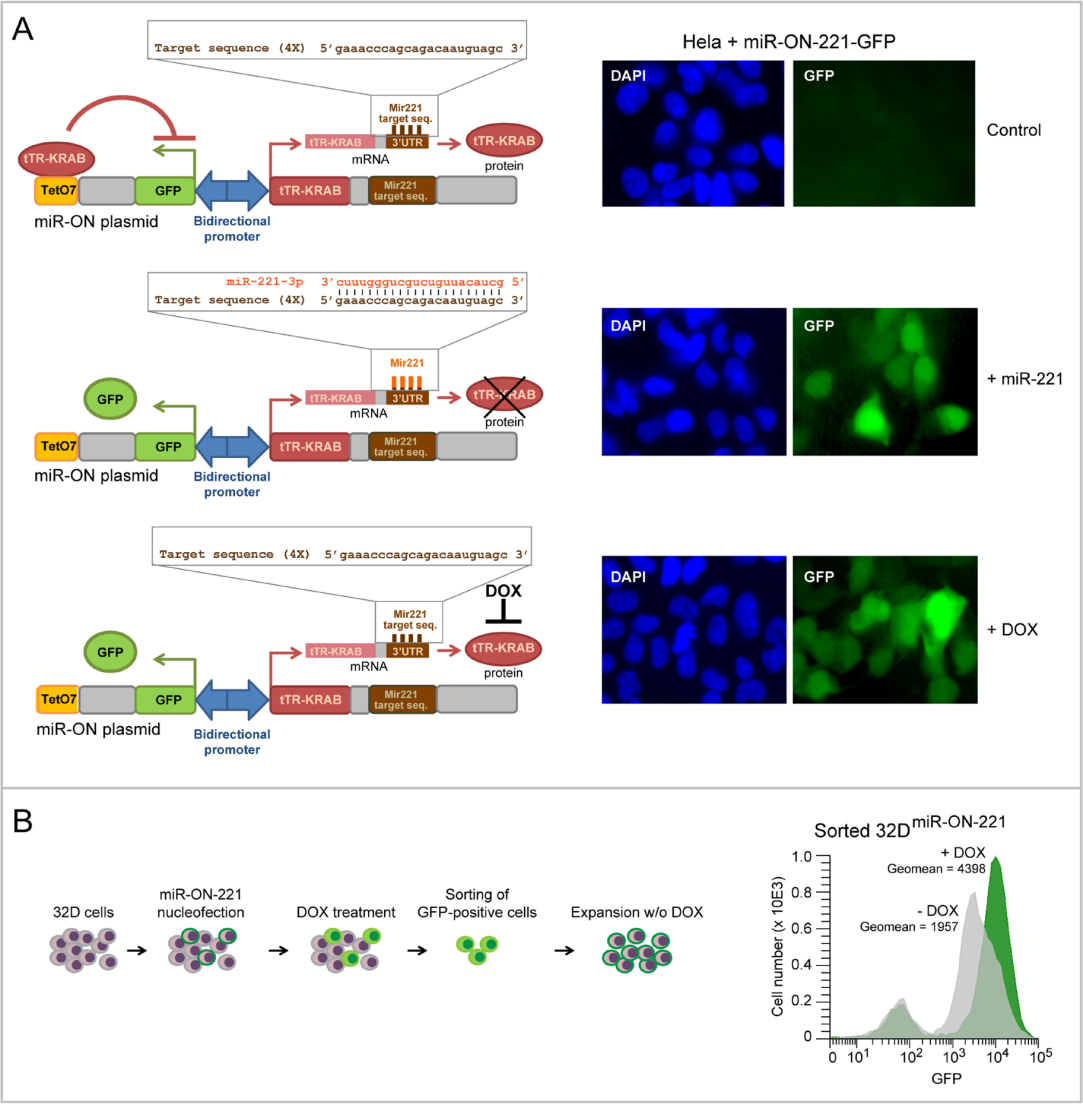
Development of 32D cells carrying the miR-ON-221 reporter plasmid to concomitantly assess miR-221 and KIT levels in single cells (**A**) The miR-ON-221 plasmid contains a bidirectional promoter that drives GFP under the control of the tetracycline operator (tetO7), as well as the tetracycline repressor-Kruppel-associated box (tTR-KRAB) carrying four tandem miR-221-target sequences in its 3′UTR. Transient transfection of miR-ON-221 in Hela cells shows that miR-ON-221 is not active (no GFP) in the absence of miR-221 (top), but is activated (presence of GFP) either by co-transfection with exogenous miR-221, which binds to its target sequences in the 3′ UTR of the tTR-KRAB repressor mRNA leading to mRNA degradation (middle), or in the presence of doxycycline (1 µg/ml, 48 h), which prevents tTR-KRAB from binding the tetracycline operator (bottom). (**B**) Scheme showing the development of the 32D^miR-ON-221^ stable line carrying the miR-ON-221 plasmid (left). Cytofluorimetric analysis of 32D^miR-ON-221^ cells shows increased GFP expression in response to DOX (1 µg/ml, 7 days), thus confirming the presence and functionality of the miR-ON-221 plasmid (right).

32D myeloblasts carrying wild-type RUNX1 grow undifferentiated in the presence of IL-3, but undergo granulopoiesis when IL-3 is replaced by GCSF [17]. To concomitantly assess both miR-221 and KIT expression level variation during granulopoiesis in individual 32D cells, we took advantage of the ‘self-contained’ miR-ON reporter plasmid [16]. This plasmid exploits two OFF switches under the control of a bidirectional promoter: a tetracycline repressor (tTR-KRAB) containing a miR-target sequence in its 3′UTR, and a GFP reporter cassette controlled by the tTR-KRAB repressor via a tetracycline operator (Tet-O). For this study we developed a miR-ON plasmid with a tTR-KRAB repressor flanked by a 3′UTR containing four sequences with perfect complementarity to miR-221 (Figure 1A, left schemes). Initial testing in Hela cells showed that the miR-ON-221 reporter was specifically activated by either co-transfection of exogenous miR-221, which leads to degradation of the tTR-KRAB repressor, or treatment with 1 µg/ml Doxycycline (DOX) (positive control), which blocks tTR-KRAB repressor binding to the TetO operator (Figure 1A, right panels). Next, we developed 32D cells stably carrying miR-ON-221 (32D^miR-ON-221^). Induction of GFP in 32D^miR-ON-221^ cells nucleofected with miR-ON-221 was detected after treatment with DOX (1 µg/ml) for 7 days. GFP-positive 32D^miR-ON-221^ cells were sorted, expanded, and tested by flow cytometry for GFP induction by DOX to confirm the presence of a functional miR-ON-221 plasmid (Figure 1B).

**Figure 2:**
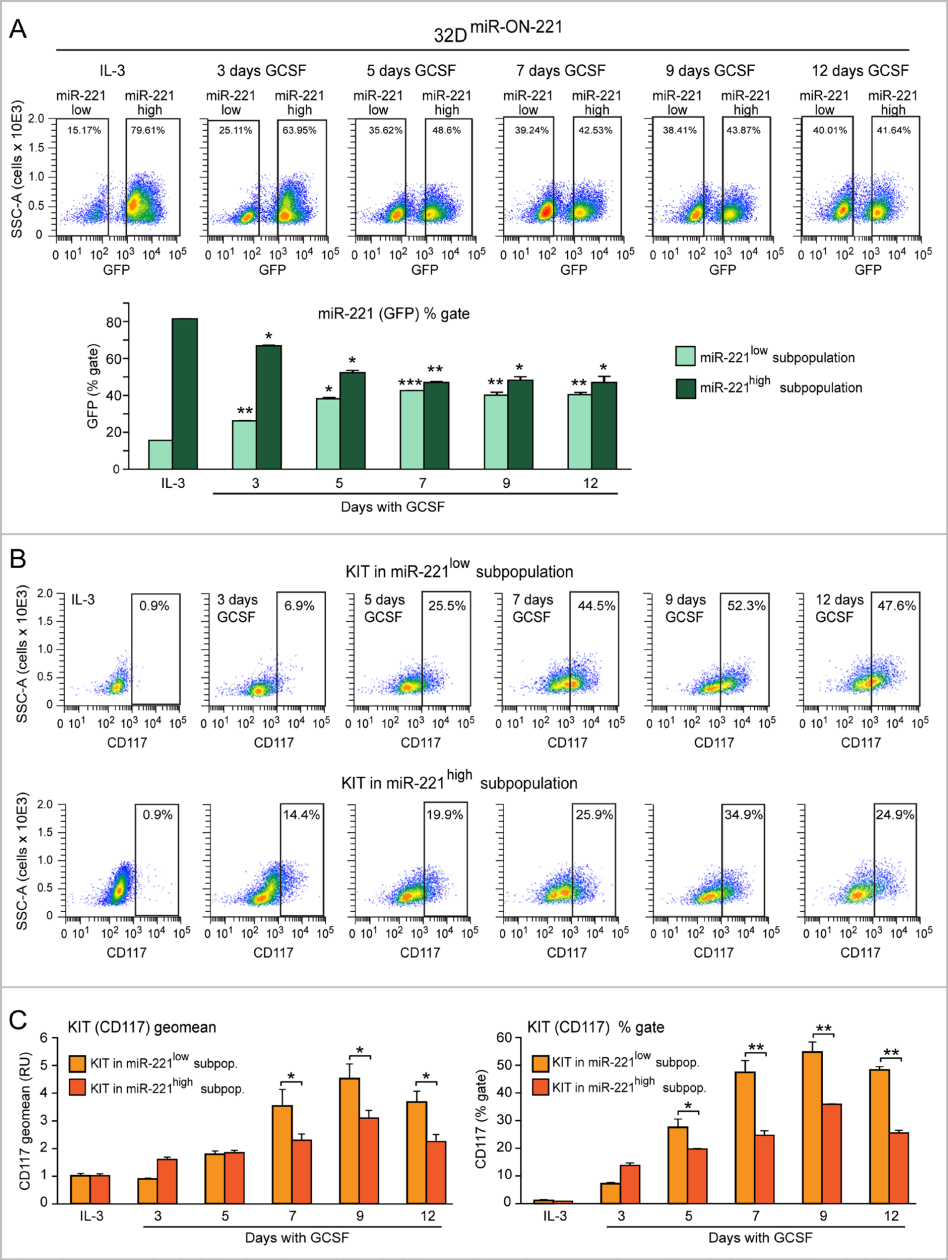
Evidence of antithetic variation of miR-221 and KIT levels in single 32D^miR-ON-221^ cells during 32D granulopoiesis (**A**) Cytofluorimetric analysis of 32D^miR-ON-221^ cells at different days after GCSF treatment (representative density plots are shown on top) shows a progressive increase in the miR-221^low^ subpopulation and a decrease in the miR-221^high^ subpopulation (assessed as GFP % gate) relative to cells grown with IL-3 (bottom). (**B** and **C**) Cytofluorimetric analysis of 32D^miR-ON-221^ cells at different days after GCSF treatment (representative density plots are shown in B) shows that KIT level, assessed either as geo mean (C, left) or % gate relative to IL-3 (C, right), is higher in the miR-221^low^ subpopulation relative to the miR-221^high^ subpopulation. *p < 0.05, **p < 0.01, ***p < 0.001.

To concomitantly assess both miR-221 and KIT expression level variation during 32D^miR-ON-221^ granulopoiesis, we analyzed GFP (for miR-221) and CD117 (for KIT receptor) by flow cytometry, first in the presence of IL-3, and subsequently in the course of a 12 day GCSF treatment. As shown in Figure 2A, top, undifferentiated 32D^miR-ON-221^ cells grown in the presence of IL-3 displayed a subpopulation of cells (∼15%) with miR-221^low^_,_ and a subpopulation of cells (∼79%) with miR-221^high^. Upon replacement of IL-3 with GCSF, the 32D miR-221^low^ cell subpopulation significantly increased from day 3 to day 12 at the expense of the 32D miR-221^high^ subpopulation (Figure 2A, bottom).

By cytofluorimetric analysis we also assessed the influence of miR-221 level variation on KIT receptor (CD117) level variation, in both 32D miR-221^low^ (Figure 2B, top) and 32D miR-221^high^ (Figure 2B, bottom) cell subpopulations. Analysis of the CD117 geomean (i.e. average CD117 level) showed that a) KIT (CD117) level significantly increased upon GCSF treatment in both miR-221^low^ and miR-221^high^ subpopulations, with a peak at day 9 (Figure 2C, left); and b) KIT (CD117) was expressed significantly more in the miR-221^low^ subpopulation relative to the miR-221^high^ subpopulation between day 7 and day 12 (Figure 2C, left). Similar differences were found when we analyzed the percent of KIT (CD117)-positive cells (relative to IL-3) in the miR-221^low^ subpopulation versus the miR-221^high^ subpopulation (Figure 2C, right).

Thus, by monitoring the dynamics of miR-221 level variation in single cells with the miR-ON-reporter strategy, we could detect that the miR-221 level is differentially expressed in 32D myeloblast subpopulations in the presence of IL-3. However, during a 12 day GCSF treatment, the 32D miR-221^low^ cell subpopulation progressively increases, while the 32D miR-221^high^ cell subpopulation progressively decreases. Consistent with the repressive role of miR-221 on KIT receptor expression, the increase of KIT level was higher in the 32D miR-221^low^ cell subpopulation relative to the 32D miR-221^high^ cell subpopulation. Based on the overall findings in the course of 32D^miR-ON-221^ granulopoiesis, the level of miR-221 and KIT vary dynamically, and antithetically, during the GCSF-induced initial myeloblast proliferation, and subsequent differentiation.

### Progressive KIT upregulation due to progressive miR-221 repression in 32D-RM8^miR-ON-221^ cells drives continuous proliferation of undifferentiated myeloblasts

**Figure 3:**
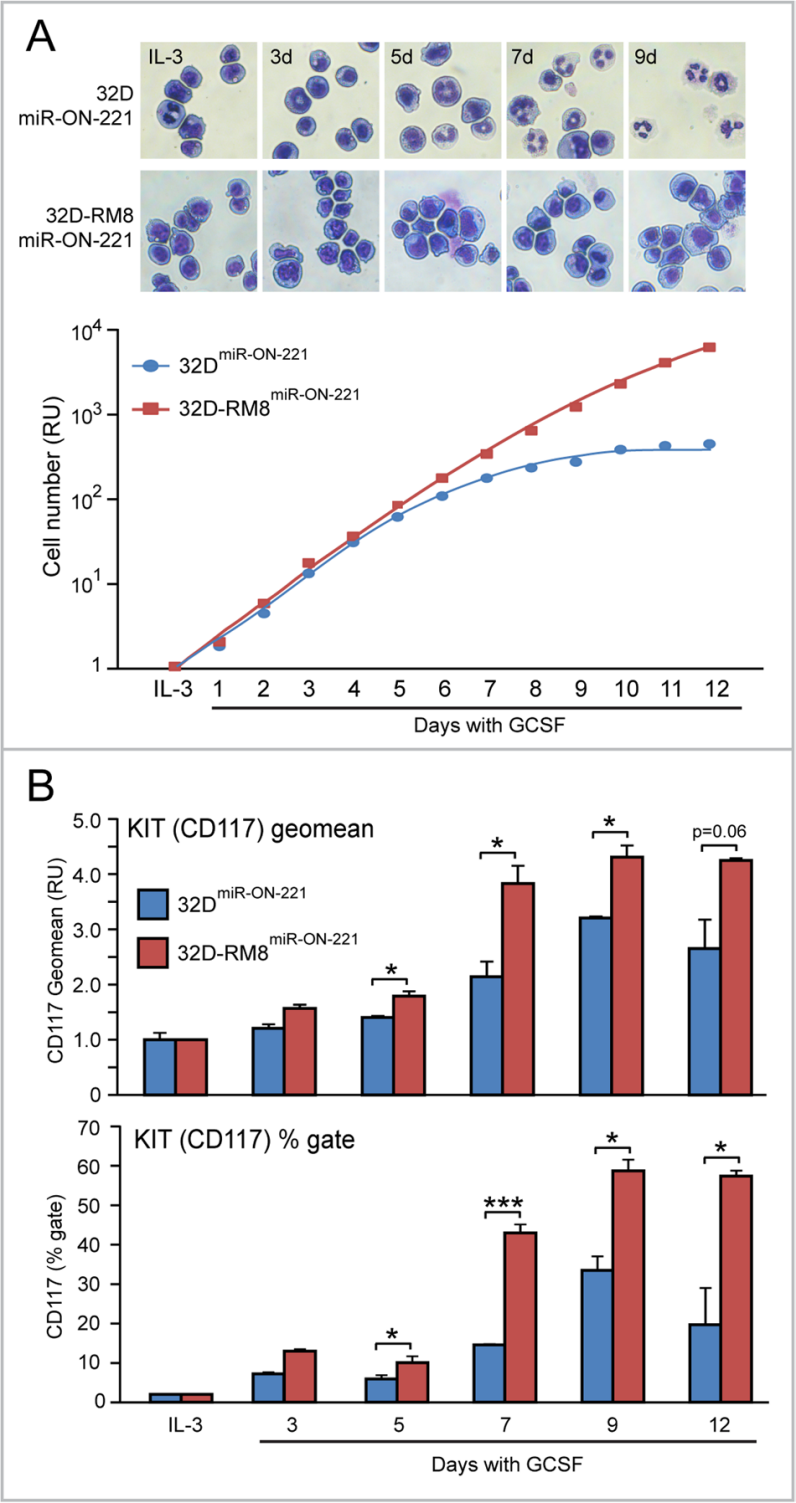
Progressive KIT upregulation in 32D-RM8^miR-ON-221^ cells drives continuous proliferation of undifferentiated myeloblasts (**A**) Giemsa staining of cytospin preparations shows that, differently from 32D^miR-ON-221^ control cells, 32D cells stably transfected with RUNX1-MTG8 and miR-ON-221 (32D-RM8^miR-ON-221^) fail to undergo granulocytic differentiation (see lack of segmented nuclei) (top). Consistently, 32D-RM8^miR-ON-221^ cells continue proliferating after day 7, whereas at this stage control cells stop proliferating (bottom). (**B**) CD117 cytoflyuorimetric analysis shows that KIT level (assessed both as geo mean, shown on top, and % gate, shown at the bottom) increases significantly more in 32D-RM8^miR-ON-221^ relative to control 32D^miR-ON-221^ during GCSF treatment. *p < 0.05, ***p < 0.001.

The t(8;21) leukemia RUNX1-MTG8 fusion protein (RM8), which interferes with wild type RUNX1 transcriptional function, by repressing miR-221 transcription, was shown to lead to KIT receptor upregulation [13]. To test in 32D single cells the effects of RM8 on miR-221-regulated KIT level variation in the presence of both IL-3 and in response to a 12 day GCSF exposure, we developed 32D-RM8 cells stably expressing miR-ON-221 (32D-RM8^miR-ON-221^). In response to a 12 day GCSF treatment, differently from 32D^miR-ON-221^ cells, 32D-RM8^miR-ON-221^ cells were unable to undergo granulocytic differentiation (see lack of segmented nuclei in Figure 3A, top), and continued proliferating also after day 5. In contrast, at this time point, the 32D^miR-ON-221^ cell proliferation curve started plateauing and diverging from the 32D-RM8^miR-ON-221^ proliferation curve (Figure 3A, bottom). Cytofluorimetric comparison of both 32D^miR-ON-221^ and 32D-RM8^miR-ON-221^ cells KIT level (CD117) – measured either as geomean (Figure 3B, top) or percent of KIT-positive cells during a 12 day GCSF exposure (Figure 3B, bottom) – showed that 32D-RM8^miR-ON-221^ cells had significantly higher KIT level (CD117) at all time points relative to control 32D^miR-ON-221^ cells.

**Figure 4:**
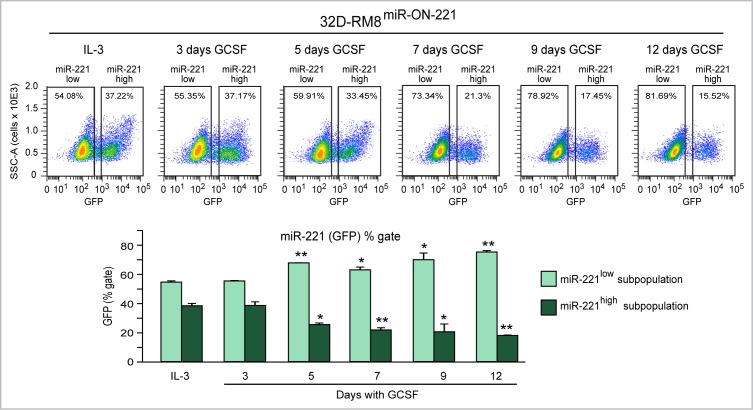
GCSF induces progressive miR-221 downregulation in 32D-RM8^miR-ON-221^ cells by expanding the miR-221^low^ subpopulation and decreasing the miR-221^high^ subpopulation Cytofluorimetric analysis of 32D-RM8^miR-ON-221^ cells at different days after GCSF treatment (representative density plots are shown on top) shows a progressive increase in the miR-221^low^ subpopulation and a decrease in the miR-221^high^ subpopulation (assessed as GFP % gate, bottom, left) relative to 32D cells grown with IL-3. *p < 0.05, **p < 0.01.

Moreover, cytofluorimetric GFP analysis of 32D-RM8^miR-ON-221^ cells grown in the presence of IL-3 detected a miR-221^low^ subpopulation (∼54%) larger than the miR-221^high^ subpopulation (∼37%) (Figure 4, top). Similar to what we detected in 32D^miR-ON-221^ cells (Figure 2A), over the 12 day GCSF treatment the 32D-RM8 miR-221^low^ subpopulation significantly increased, while the 32D-RM8 miR-221^high^ subpopulation significantly decreased (Figure 4, bottom).

**Figure 5:**
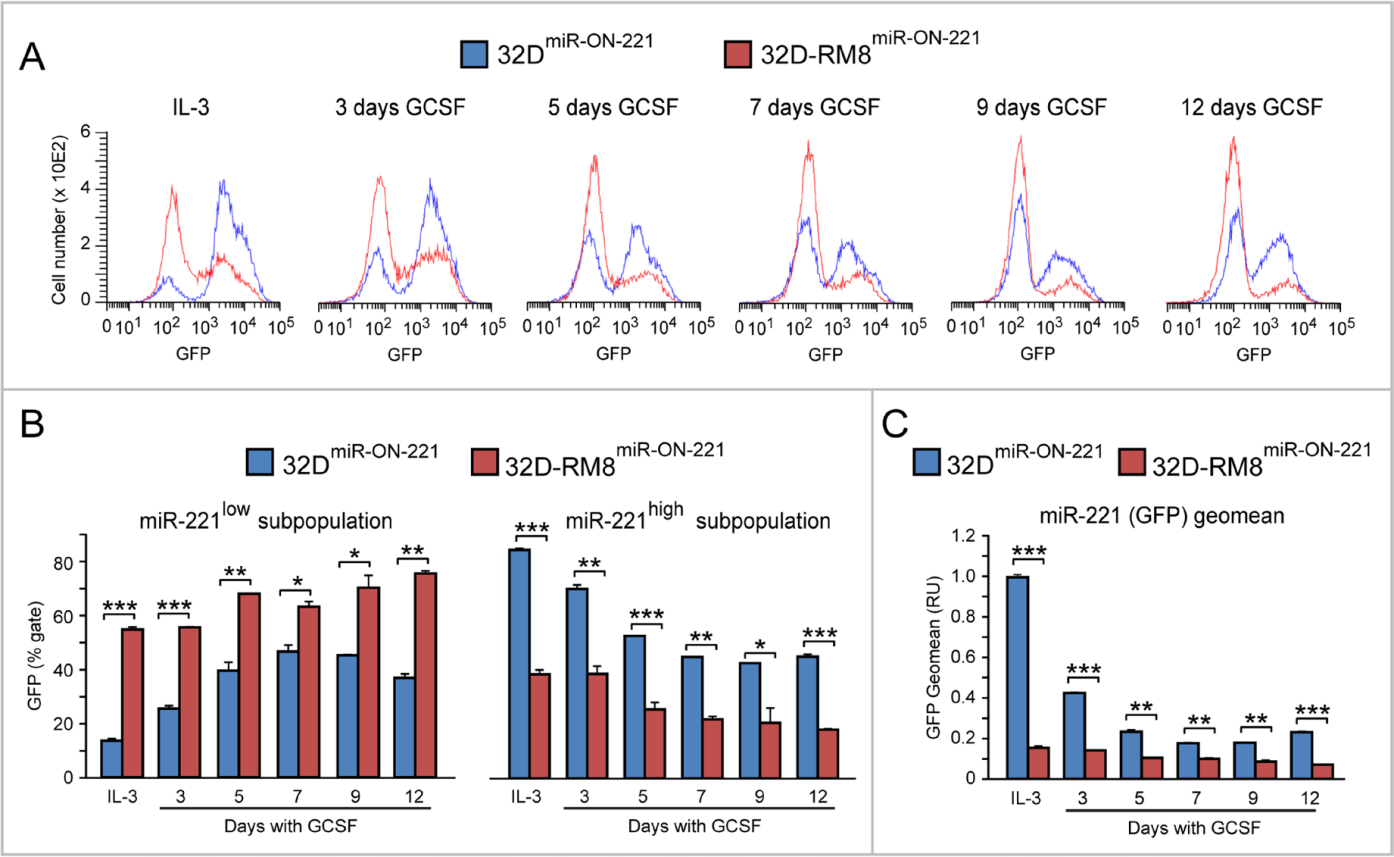
GCSF-induced miR-221 downregulation is exacerbated in 32D-RM8^miR-ON-221^ cells relative to 32D^miR-ON-221^ cells (**A**–**B**). Cytofluorimetric analysis of 32D-RM8^miR-ON-221^ vs 32D^miR-ON-221^ at different days after GCSF treatment (representative histogram plots are shown in A) shows that the miR-221^high^ subpopulation is larger (B, left), while the miR-221^low^ subpopulation is smaller (B, right), in 32D-RM8^miR-ON-221^ vs. 32D^miR-ON-221^ cells throughout GCSF treatment. (**C**) These single cell differences translate into an overall decrease of miR-221 (assessed as GFP geo mean) in 32D-RM8^miR-ON-221^ vs. 32D^miR-ON-221^ cell populations. *p < 0.05, **p < 0.01, ***p < 0.001.

Interestingly, when we compared the miR-221 level in 32D-RM8^miR-ON-221^ vs 32D^miR-ON-221^ cells by flow cytometry (Figure 5A), it was apparent that the reduction of miR-221 in response to GCSF was more drastic in 32D-RM8^miR-ON-221^ (Figures 5B and 5C). Specifically, the miR-221^low^ subpopulation was larger (Figure 5B, left) in 32D-RM8^miR-ON-221^ than in 32D^miR-ON-221^ cells, while the miR-221^high^ subpopulation was smaller (Figure 5B, right), both under IL-3 conditions, and during the 12 day GCSF exposure. The different size of the miR-221^low^ and miR-221^high^ subpopulations translated into a lower overall miR-221 level (GFP Geomean) in 32D-RM8^miR-ON-221^ vs 32D^miR-ON-221^ cells (Figure 5C).

Overall, our findings show that the RM8 mutant, which interferes with wild type RUNX1 transcriptional function, by progressively lowering the already low miR-221 level in 32D-RM8^miR-ON-221^ single cells, exacerbates the GCSF-induced proliferation potential of undifferentiated myeloblasts by progressively increasing single cell KIT level.

## DISCUSSION

Due to the high heterogeneity of both normal and cancer cells, one of the current challenges of biology is to assess how dynamic molecular changes in individual cells can collectively affect biological processes in a cell population. In this study we used the miR-ON reporter strategy [16], which enables to monitor the temporal dynamics of miRNAs in single cells. Using this strategy, here we show that it was possible to monitor the variation of wild type RUNX1 transcriptional regulation of miR-221-regulated KIT level and how this variation translates into different individual myeloblast cell fate decisions. This, collectively, contributes to dynamic cell population changes.

**Figure 6:**
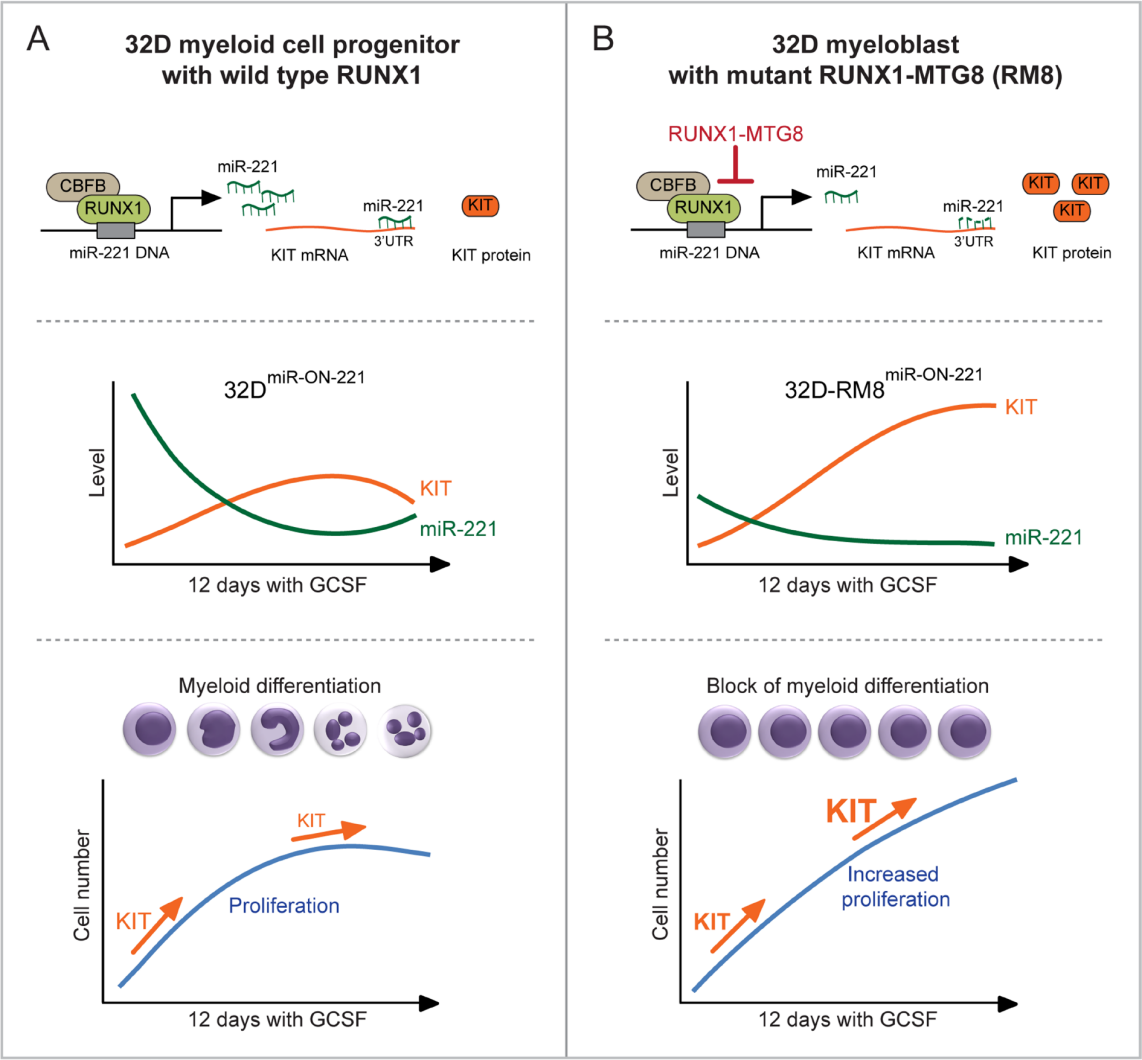
Heterogeneous single cell functional plasticity of the RUNX1-miR-221-KIT axis translates into myeloblast decisions of normal and aberrant granulopoiesis (**A**) In 32D^miR-ON-221^ cells with functional RUNX1-miR-221-KIT axis (top), GCSF leads to opposite variation of miR-221 and KIT levels in single cells (middle). MiR-221-regulated KIT level initially increases to support the expansion of the pool of progenitor myeloblasts, but it decreases at later stages of GCSF-induced granulopoiesis to allow terminal granulocytic differentiation (bottom). (**B**) In 32D-RM8^miR-ON-221^ cells, expression of RUNX1-MTG8, by interfering with RUNX1 function, transcriptionally represses the transcription of miR-221, thus leading to KIT upregulation (top). GCSF treatment decreases the already low miR-221 level, resulting in further upregulation of KIT (middle). In turn, upregulation of KIT sustains proliferation of undifferentiated myeloblasts even at the end of the 12 day GCSF treatment (bottom).

Using 32D^miR-ON-221^ cells expressing wild type RUNX1, and stably transfected with the miR-ON-221 reporter, we were able to monitor the temporal dynamics of level variation of both miR-221 and its target, KIT receptor (Figure 6A, top panel), in single cells in response to a 12 day GCSF cytokine exposure. MiR-221 level variation ranging from high to low (measured as GFP) in 32D^miR-ON-221^ cells translated into opposite level variation of KIT (measured as CD117) (Figure 6A, middle panel). At initial stages of normal 32D^miR-ON-221^ granulopoiesis in response to a 12 day GCSF exposure, the KIT level was markedly high to support the expansion of the pool of undifferentiated myeloblasts, but it decreased at later stages to allow terminal granulocytic differentiation (Figure 6A, bottom panel).

In order to assess the single cell level variation of miR-221-regulated KIT receptor expression during the progressive expansion of undifferentiated myeloblasts expressing the t(8;21) RUNX1-MTG8 (RM8) mutant, we used 32D-RM8^miR-ON-221^ cells. The RM8 mutant, by interfering with RUNX1, transcriptionally represses miR-221 transcription, thus leading to KIT upregulation (Figure 6B, top panel). During a 12 day GCSF exposure, the already low miR-221 level (measured as GFP) significantly decreased, while the KIT receptor level significantly increased (Figure 6B, middle). This is consistent with proliferation of undifferentiated 32D-RM8^miR-ON-221^ myeloblasts (Figure 6B, bottom). In this respect, it is interesting to note that miR-221 and KIT level variation during GCSF treatment showed a divergent trend in 32D-RM8^miR-ON-221^ cells (i.e. miR-221 continued to decrease, while KIT continued to increase even after 12 days) (Figure 6B, middle). In contrast, there was a convergent trend in 32D^miR-ON-221^ cells (i.e. miR-221 tended to increase, while KIT tended to decrease, at the end of GCSF treatment) (Figure 6A, middle). Deregulation of the miR-221-KIT axis could be particularly relevant in the context of leukemia with KIT receptor activating mutations [18–20]. Repression of miR-221 by RM8 in leukemia cells with a KIT activating mutation is expected to exacerbate proliferation relative to RM8-positive leukemia cells with a non-mutated KIT.

In leukemia the single cell functional plasticity of the RUNX1-miR-221-KIT axis that regulates normal proliferation and differentiation can be undermined not only by the RM8 fusion protein, but also by genetic mutations, including other non-random cytogenetic rearrangements affecting RUNX1 or CBFB [21–23]. Indeed, we previously reported that the fusion protein CBFB-MYH11, resulting from the inv(16) leukemia chromosome inversion, induced miR-221 downregulation, and consequent KIT upregulation, as the RUNX1-MTG8 fusion protein did [13]. By using the miR-ON system it is possible to test whether different genetic factors affecting the CBF subunits lead to a similar (or different) single cell deregulation of the miR-221-KIT axis. Moreover, there are other factors that can dynamically affect RUNX1 level by interfering with RUNX1 transcriptional function. One of these factors is miR-17, which regulates, and it is regulated by RUNX1 itself [24]. Overexpression of miR-17,by reducing RUNX1 level, can mimic the single cell effects of the RM8 leukemia genetic mutation [13]. In addition, recent findings in another cell context indicate that many other miRNAs targeting RUNX1 (e.g. miR-23b, miR-205, and miR-375) can play a role in determining the temporal variation of RUNX1 level during tumorigenesis [25]. Thus, the miR-ON system could be used to detect the hierarchical temporal dynamics of miRNAs regulating RUNX1 (e.g. miR-17) and RUNX1-regulated miRNAs, including miR-221 and other KIT-regulating miRNAs (e.g. miR-193a) [26, 27].

It is noteworthy that RUNX1 also controls the transcription of miRNAs critical for myeloid differentiation, such as miR-223 [28, 29]. Indeed, we found that miR-223 is transiently upregulated during GCSF-induced granulopoiesis of 32D cells with wild type RUNX1, but its upregulation is counteracted by stable expression of RM8 (Supplementary Figure 1). Thus, on one hand RM8 leads to increased KIT-mediated proliferation by downregulating miR-221, and on the other hand it counteracts miR-223-mediated granulocytic differentiation. By using the miR-ON system we could monitor the temporal modulation of these two miRNAs in single cells with either wild type or mutant RUNX1.

Expanding our knowledge of miRNA-induced dynamic biological changes, by using tools like the miR-ON reporter system, can have relevant translational implications for leukemia detection and treatment. For instance, concomitant detection of miR-221 downregulation and KIT upregulation could identify cells with a defective RUNX1-miR-221-KIT axis due to different factors that interfere with RUNX1 transcriptional function. In addition, miRNAs are emerging as promising targets for cancer therapy. Indeed, compounds that either inhibit or mimic miRNAs in cancer are being evaluated both in pre-clinical and clinical studies [30]. Thus, strategies to modulate miRNA dynamics should enable us to target leukemia by coordinately inhibiting pro-proliferative signaling pathways and inducing differentiation signaling pathways.

## MATERIALS AND METHODS

### Cells and culture conditions

HeLa cells were cultured in DMEM medium (Thermo Fisher, Waltham, MA) supplemented with 10% FBS (Thermo Fisher). 32D cells expressing wild type RUNX1 and GCSF receptor [17] and 32D cells carrying the RUNX1-MTG8 fusion protein (32D-RM8) [13], and derived cell lines were cultured in RPMI 1640 (Thermo Fisher) medium supplemented with 10% heat-inactivated (HI) fetal bovine serum (FBS) (Thermo Fisher) and 1 ng/ml murine IL-3 (Peprotech, Inc., Rocky Hill, NJ). Cells were counted daily with a Coulter Particle Counter (Beckman Coulter, Brea, CA) and diluted to a density of 2 × 10^5^ cells/ml daily.

### Development and validation of the miR-ON-221 plasmid

The ‘self-contained’ miR-ON plasmid was kindly provided by Dr. Luigi Naldini, San Raffaele University, Milan, Italy [16]. The pMA-RQ-Bb-miR-221 plasmid, containing four tandem miR-221-3p-target sequences (5′-gaaacccagcagacaauguagc-3′) flanked by KpnI and XbaI restriction sites, was synthesized by Thermo Fisher. In order to obtain the miR-ON-221 plasmid, the four miR-221-target sequences were cut out from the pMA-RQ-Bb-miR-221 plasmid and sub-cloned into the KpnI and XbaI restriction sites (in the 3′UTR of the tTR-KRAB repressor) of the ‘self-contained’ miR-ON plasmid. The miR-ON-221 plasmid was verified by sequencing with a WPRE sense primer (5′-tgttgggcactgacaatttcc-3′).

In order to preliminarily test miR-ON-221 functionality, HeLa cells were seeded in 24-well plates and transiently transfected with 200 ng of miR-ON-221 construct by using Lipofectamine 2000 (Thermo Fisher). Twenty-four hours after transfection the medium was replaced with DMEM with or without 1 µg/ml doxycycline. Two days after doxycycline treatment, cells were fixed with 4% paraformaldehyde, permeabilized with 0.2% Triton-X 100, counterstained with DAPI, and analyzed with a fluorescence microscope. Hela cells were also transiently co-transfected with 200 ng miR-ON-221 and 400 ng of either a plasmid expressing exogenous miR-ON-221 or the empty vector. After 48 h, GFP fluorescence was analyzed with a fluorescence microscope to test if miR-221 was able to activate the miR-ON-221 plasmid.

### Development of miR-ON-221 32D cell lines

To develop 32D cell lines stably expressing the miR-ON-221 plasmid, both 32D cells and 32D-RM8 cells were nucleofected with the miR-ON-221 plasmid using an Amaxa nucleofector (Lonza, Basel, Switzerland). The nucleofected cells were treated with 1 µg/ml doxycycline for seven days to induce GFP expression. GFP-positive cells (i.e. cells carrying miR-ON-221) were sorted by using a FACS Aria I cell sorter (BD, Franklin Lakes, NJ). Approximately 10^4^ doxycycline-treated cells were sorted, using non-treated cells as a control to set the GFP-positive gate. The GFP-positive cells were collected and expanded in 32D growth medium without doxycycline and further tested for miR-ON functionality based on GFP induction after treatment with 1 µg/mL doxycycline for seven days.

### Induction of 32D granulopoiesis by GCSF

To induce 32D cell granulopoiesis, IL-3 was replaced with 10 ng/ml human GCSF (Amgen, Thousand Oaks, CA). Cells were grown in culture for twelve days at a density of 2 × 10^5^ cells/ml, and counted daily to obtain a growth curve to assess proliferation dynamics. Differentiation was assessed microscopically on Giemsa-stained cytospin preparations. Cells were spun onto glass slides using a Cytospin 3 machine (Marshall Scientific, Hampton, NH), allowed to dry, and fixed with methanol for ten minutes. Slides were immersed in Giemsa Stain Modified Solution (Sigma-Aldrich, St. Louis, MO) diluted 1:20 in double-distilled water for forty-five minutes, rinsed with distilled water, and imaged using a light microscope.

### Flow cytometry analysis of miR-221-regulated KIT expression in single cells

KIT expression was assessed by cytofluorimetric analysis of the CD117 antigen, while miR-221 expression was assessed based on activation of the GFP reporter miR-ON-221. To concomitantly measure KIT (CD117) and miR-221 (GFP) expression in 32D^miR-ON-221^ and 32D-RM8^miR-ON-221^ cells, 0.5–1 × 10^6^ cells were resuspended in 95 µl blocking buffer (PBS+0.5% BSA) and incubated with a PE-CD117 antibody (Miltenyi Biotec, San Diego, CA) for twenty minutes in the dark on ice. Unstained cells were used as a negative control. After incubation, cells were washed with blocking buffer, resuspended in 500 µl blocking buffer, and analyzed by flow cytometry on an LSR Fortessa cytometer (BD Biosciences). Data were analyzed using WinList software. MiR-221 expression in the entire cell population was evaluated by calculating the GFP geomean. MiR-221^high^ and miR-221^low^ subpopulations were gated based on the presence of two distinct subpopulations with different GFP fluorescence in cells grown with IL-3; these gates were applied to each time point with GCSF to calculate the percentage of cells with low or high miR-221 expression. After GCSF treatment, KIT expression at different time points was calculated either as PE geomean of the total cell population, or as percent gate of KIT-positive cells relative to IL-3 (set as ∼ 1%). KIT expression in the miR-221^high^ and miR-221^low^ subpopulations was assessed either by measuring the PE geomean of the two subpopulations, or as percent gate of KIT-positive cells relative to IL-3 (set to ∼ 1%) within each subpopulation. The KIT geo mean of cells grown with GCSF was shown relative to the geo mean of cells grown with IL-3 (set as 1). Statistical significance was calculated by using the Student’s *t-*test.

## SUPPLEMENTARY MATERIALS FIGURE


