# Supplementary material for: MiR-221-regulated KIT level by wild type or leukemia mutant RUNX1: a determinant of single myeloblast fate decisions that – collectively – drives or hinders granulopoiesis
Source: Oncotarget. 2017 Sep 23;8(49):85783–93. doi: 10.18632/oncotarget.21266 (PMC5689646; doi:10.18632/oncotarget.21266)
Supplement: Supplementary file 1 [file oncotarget-08-85783-s001.pdf]

MiR-221-regulated KIT level by wild type or leukemia mutant RUNX1: a determinant of single myeloblast fate decisions that – collectively – drives or hinders granulopoiesis

SUPPLEMENTARY MATERIALS

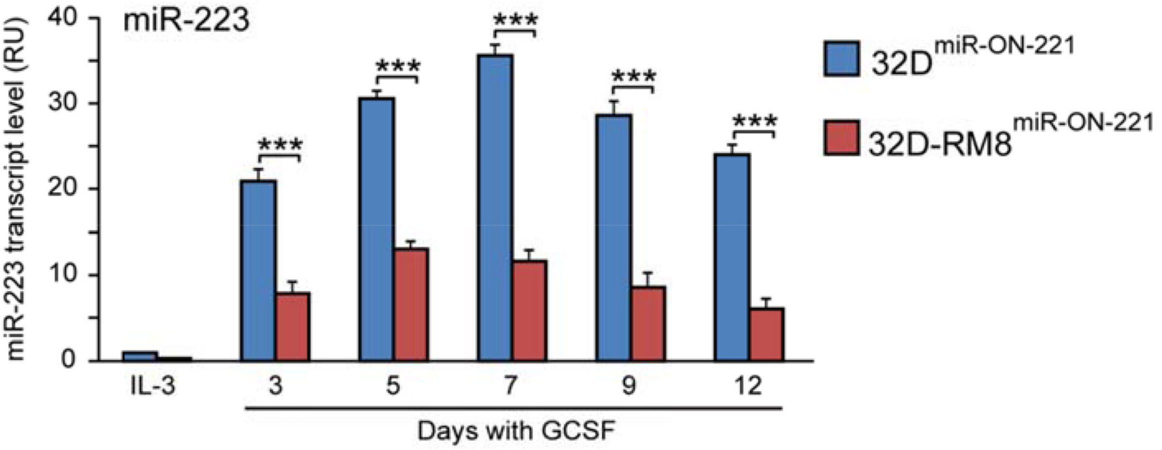

Supplementary Figure 1: Stable expression of RUNX1-MTG8 (RM8) affects the temporal modulation of miR-223 in 32D cells. qRT-PCR shows that miR-223 transcript level is dynamically modulated during GCSF-induced granulopoiesis of 32D cells carrying wild type RUNX1 ($32D^{miR\text{-}ON\text{-}221}$, blue bars). Stable expression of RM8 in $32D\text{-}RM8^{miR\text{-}ON\text{-}221}$ significantly reduces miR-223 level at all stages of GCSF-induced granulopoiesis. ***$p < 0.001$.
